# Seeking to be seen as legitimate members of the scientific community? An analysis of British American Tobacco and Philip Morris International’s involvement in scientific events

**DOI:** 10.1136/tc-2022-057809

**Published:** 2023-02-03

**Authors:** Britta Katharina Matthes, Alice Fabbri, Sarah Dance, Louis Laurence, Karin Silver, Anna B Gilmore

**Affiliations:** 1 Department for Health, University of Bath, Bath, UK; 2 Department of Psychology, University of Bath, Bath, UK

**Keywords:** tobacco industry, denormalization, global health

## Abstract

**Introduction:**

For decades, tobacco companies manipulated and misused science. They funded and disseminated favourable research and suppressed research that showed the harms of their products, deliberately generating misinformation. While previous work has examined many of the practices involved, their engagement in scientific events has so far not been systematically studied. Here, we examine the involvement of British American Tobacco (BAT) and Philip Morris International (PMI) in scientific events, including conferences, symposia and workshops.

**Methods:**

Our analysis involved two steps. First, we collected all available data PMI and BAT provided on their websites to identify events. Second, we extracted information about the nature of tobacco industry involvement from event websites and materials.

**Results:**

We identified 213 scientific events that BAT and/or PMI representatives attended between April 2012 and September 2021. Most events took place in high-income countries in Europe and North America. They covered a diverse range of fields, including toxicology (n=60, 28.1%), medicine (n=25, 11.7%), biology (n=24, 11.3%), chemistry (n=23, 10.8%) and aerosol science (n=18, 8.5%), as well as dentistry (n=9, 4.2%), pharmaceutical science (n=8, 3.8%) and computing (n=8, 3.8%). We identified 356 posters provided by BAT and PMI that linked to 118 events (55.4%) as well as 77 presentations from 65 events (30.5%). Industry involvement through sponsorship (nine events), exhibition (three events) or organising committee (one event) was rare.

**Conclusion:**

BAT and PMI representatives attended a large number and wide range of scientific events. Given that scientific events could be a crucial platform for building connections in the scientific sphere and disseminating industry’s messages, this work highlights the importance of denormalising the tobacco industry’s involvement in scientific events.

**WHAT IS ALREADY KNOWN ON THIS TOPIC**

- The tobacco industry seeks to be perceived as a legitimate player in the scientific community and policymaking.
- The tobacco industry has a track record of manipulating evidence, creating disinformation and concealing conflicts of interests, prioritising profits over science.
- Scientific events such as conferences and workshops could serve as an important platform for the tobacco industry to spread its messages. Little is known about the tobacco industry’s involvement in scientific events.

**WHAT THIS STUDY ADDS**

- British American Tobacco and Philip Morris International records suggest that between 2012 and 2021 their representatives attended a large number of scientific events, mostly in high-income countries, covering a wide range of fields and frequently presented posters or delivered presentations.
- Tobacco industry presence as sponsor, exhibitor or within the organising committee was rare with some notable exceptions.

**HOW THIS STUDY MIGHT AFFECT RESEARCH, PRACTICE OR POLICY**

- Given the industry’s history of scientific misconduct and generating disinformation, event organisers should consider the implications of tobacco industry participation at their events and the adoption of policies precluding its involvement.
- The public health community should raise awareness about tobacco industry presence at scientific events and its implications.

## Introduction

The tobacco industry has for decades attempted to create and maintain an image of scientific credibility. Driven by its desire to maximise profits, it has used science to obscure the harms caused by its products and avoid unfavourable regulation and litigation.^1^ Internal industry documents released through whistleblowers and litigation revealed that the industry was aware of the dangers of smoking and secondhand smoke and the addictiveness of nicotine, but instead of acting to prevent further harm, tobacco companies sought to hide it.^2–4^ Among other things, they invested large sums of money in funding and disseminating research which claimed tobacco does not cause cancer,^5^ intentionally concealed the potential toxicity of its products^6^ as well as the addictive nature of nicotine^7^ and created an international programme of scientific consultants to shape public opinion on secondhand smoke.^8^ This disinformation led to significant delays in addressing tobacco’s harm.

To address the overwhelming evidence of the tobacco industry’s scientific misconduct, several measures were taken. For example, the 1998 Master Settlement Agreement ordered the dissolution of three tobacco industry research bodies (Council for Tobacco Research (formerly Tobacco Industry Research Committee), Tobacco Institute, Centre for Indoor Air Research).^9^ The 2006 Kessler verdict banned US-based tobacco companies from reconstituting the form or function of these bodies given their role in fraudulently deceiving the American public.^9^ Several reputable health journals, including some BMJ journals^10^ (such as *Tobacco Control* 11) and PLoS journals,^12^ will no longer consider tobacco industry-sponsored research; research funders such as Cancer Research UK,^13^ the Norwegian Cancer Society,^14^ the Irish Health Research Board^15^ and the Wellcome Trust^16^ adopted policies to protect the work they fund from association with tobacco industry interests; and several universities have policies precluding acceptance of tobacco industry research funding^17^ with some also now rejecting funding from the Foundation for a Smoke-Free World (FSFW), a recently established industry scientific front group.^18^ Some conference organisers, including the World and European Conferences on Tobacco or Health^19–21^ and, more recently, the Society for Research on Nicotine and Tobacco (SRNT)^22^ and the World Heart Federation,^23^ have adopted policies seeking to exclude industry from their events.

However, at a time when the tobacco companies are launching new products which are seen as crucial to their future and yet the safety of these products remains uncertain, the industry is again actively engaging in and funding research.^24^ Companies conduct research internally but also fund others, and in 2017 Philip Morris International (PMI) set up the FSFW with close to US$1 billion funding which claims to "support medical, agricultural and scientific research".^25^ Furthermore, much of the evidence relating to the health effects and efficacy of newer products is affiliated with the tobacco industry,^26^ and researchers have raised concerns regarding industry’s research practices: for instance, reporting of tobacco industry-sponsored trials examining e-cigarettes was found to be neither complete nor transparent^27^ and a strong association was found between authors’ financial conflicts of interests and tobacco and e-cigarette industry-favourable findings.^28^ Conduct and reporting of interventional clinical trials studying the effects of heated tobacco products, most of which were affiliated with the industry, were assessed as inappropriate for determining the impact of such products on public health.^24^ Researchers have also strongly challenged the FSFW’s claims of independence and legitimacy.^29 30^ There has also been criticism of the way the industry communicates with regulators^31^ and its customer base,^32 33^ which contrasts with the material it directs to its shareholders,^34^ and there has been scrutiny of scientific publishing practice, exposing flaws such as failure of industry-funded researchers to disclose conflicts of interest and efforts to circumvent journal policies to exclude the tobacco industry by submitting under third parties.^9 25 29 30 35^

Scientific events like conferences, symposia and workshops, where ideas and findings are presented and discussed and networks are built, play a key role in the scientific process.^36^ Internal industry documents reveal that as early as the 1950s, the tobacco industry identified conferences as places where it could manufacture controversy among scientists, controversy that it could later publicise in the media.^37^ Ever since, as well as creating their own conferences, tobacco companies have sponsored and infiltrated events such as public health conferences, where there is an opportunity to influence discussions on secondhand smoke.^38–40^ The German Association of Cigarette Industry (Verband der Cigarettenindustrie) worked on ‘influencing publications’ presented at the 4th World Conference on Smoking and Health which focused on the cost of smoking.^41 42^ At the 6th World Conference on Smoking and Health in Japan in 1987, Japan Tobacco had around 40 scientists presenting "neutral" papers to "change the very nature and tone" of the conference.^38 43^ In the early 1990s, British American Tobacco (BAT) and PMI sought to undermine the 8th World Conference on Tobacco or Health in Argentina by creating a distraction through the use of media- and science-focused campaigns.^8^

In the context of e-cigarettes and heated tobacco products, conferences were again identified as key arenas by the industry, this time to build its own credibility rather than undermine the credibility of others: according to PMI’s 2014 ‘Reduced Risk Products Briefing’^44^ released by Reuters,^31^ in addition to publications, policy papers and research institutions, posters are listed as scientific engagement "tools". No study has systematically assessed the tobacco industry’s involvement in scientific events in recent years. This paper therefore aims to help understand the scope and type of industry involvement and asks:

- What scientific events has the tobacco industry attended in recent years?
- What is the extent and nature of its involvement in these events?

## Methods

We used a two-step approach to data collection and analysis, starting with the information available on industry websites and then supplementing with information from event websites and materials.

### Step 1: industry data

#### Data sources

This study is based on the publicly available information downloaded from the Science websites of BAT and PMI, the two companies with the largest share in the global cigarette market (excluding the Chinese National Tobacco Corporation).^45^ Both companies had a section dedicated to conferences on their websites where they listed the events attended.

All available event information and materials were manually downloaded from the Science websites^46 47^ of the companies^48 49^ in December 2020 (PMI) and March 2021 (BAT). BAT and PMI Science websites were checked for updates in September 2021 following which 19 events (all PMI) were added.

The conference information (date, location, title) was entered into an Excel spreadsheet. In the case of PMI entries, a link to the event website was also provided.

#### Selection of events

We excluded those entries where the link to the tobacco company’s web page with details of the event did not work (PMI, n=2). This was because we were unable to identify the event based on the information listed (year and title).

We screened the remaining events and excluded: (a) events that were cancelled and (b) events where we were unable to determine the conference organiser, and hence the nature of the conference. We also removed duplicates (ie, the same event appearing in BAT and PMI records).

To be included, an event had to (a) not be run by industry and (b) not be a multistakeholder forum with industry partners (eg, Cooperation Centre for Scientific Research Relative to Tobacco Congress,^50^ Global Forum on Nicotine,^51^ Global Tobacco & Nicotine Forum^52^).

In order to check whether the event met our inclusion criteria we used the data retrieved from the company websites and searched online for the event. One author (BKM) assessed all the events for inclusion, a second author (AF) double-checked. In case of disagreement, another author (SD) triple-checked. Any disagreement was discussed until consensus was reached.

#### Coding of events

The included events were coded according to the following variables: (1) year, (2) location (by host country WHO region and income economy group), (3) conference presentations and posters available on company websites, (4) field and (5) type of organiser/host. In the case of 1–3, the coding was done by one author and double-checked by another author. For 4 and 5, pairs of authors (AF/LL and BKM/AF) used an inductive approach to develop the coding frameworks.

### Step 2: event websites and materials

To supplement the information about the event available on the company websites, we developed a coding manual for retrieving additional information on the included events (see online supplemental table 1). In November and December 2021, we searched the website of the conferences and collected information regarding BAT and/or PMI involvement in the form of (a) sponsoring the event, (b) being an exhibitor and (c) being part of the organising committee. We included events where information was provided in a language spoken by the research team members (English, German, Italian or Spanish) and where information on one or more of the above-mentioned criteria was publicly available.

10.1136/tc-2022-057809.supp1 Supplementary data

Three authors (AF, BKM, SD) piloted, subsequently refined the manual and coded all included events. A quarter of the events were double-coded. The agreement was 70.4% (38/54). The double coding only yielded a small amount of additional information in one instance (1.9%, 1/54).

## Findings

**Figure 1:**
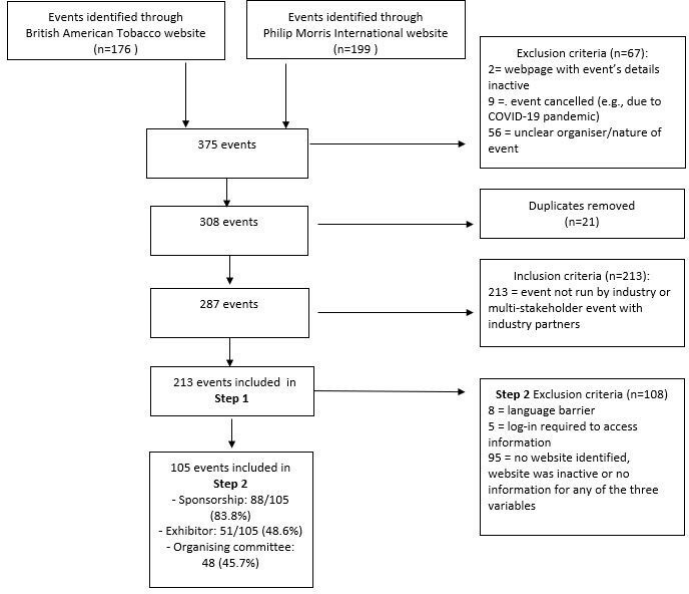
Flow diagram.

As figure 1 shows, 375 events were identified from BAT and PMI websites with 213 events included for the first part of the analysis (step 1) once the exclusion and inclusion criteria had been applied and duplicates removed. After searching event websites or materials for details of tobacco industry involvement in event sponsorship, exhibition and organising committee, 108 events were excluded due to insufficient information, leaving 105 events included in the second step of the analysis.

### Findings from step 1: tobacco industry data

#### Year

**Figure 2:**
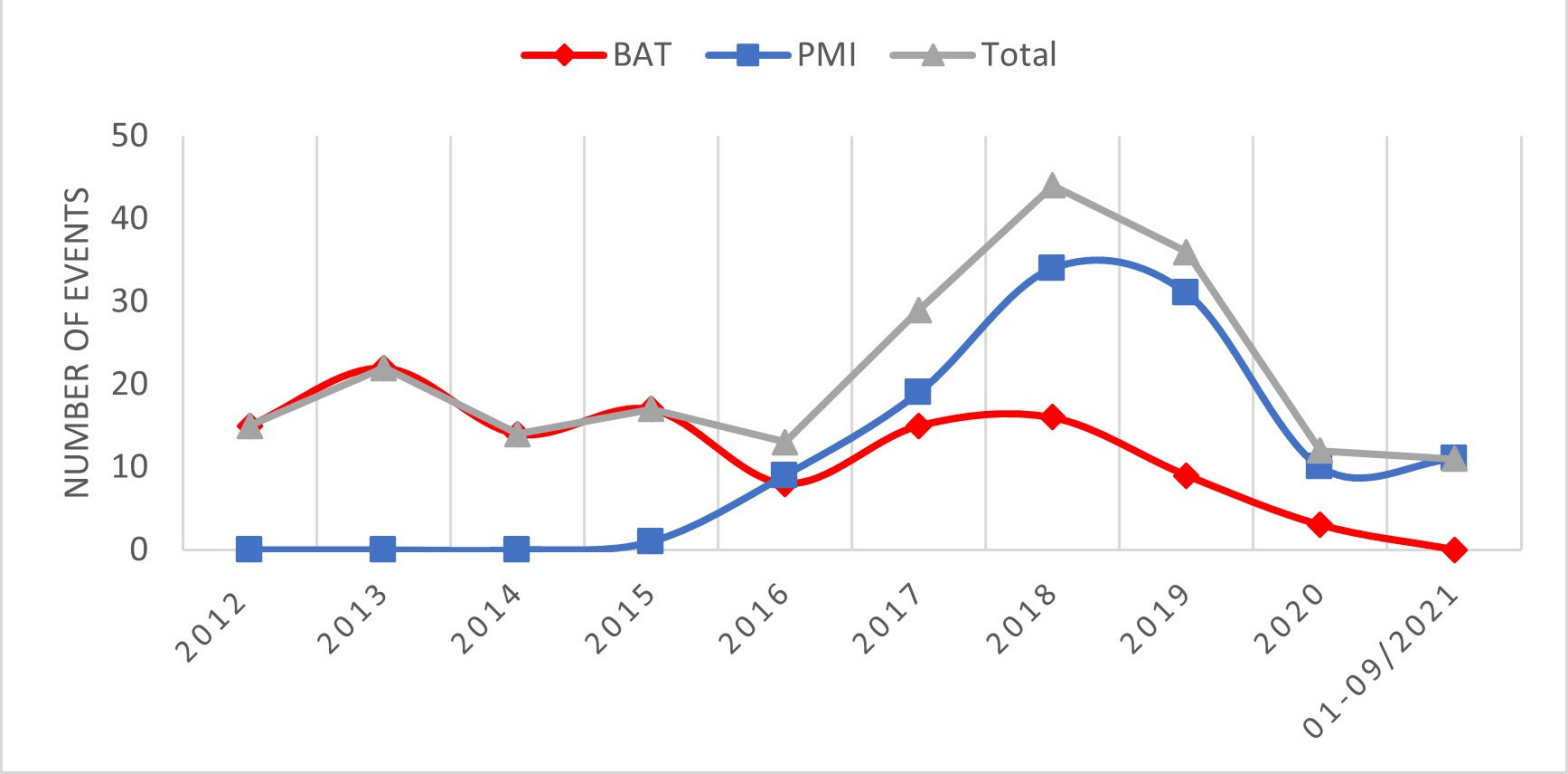
Number of events by year (21 events were attended by both British American Tobacco (BAT) and Philip Morris International (PMI) which is why the numbers per year do not always add up to the total).

The earliest event was in April 2012 and the last one was in September 2021. In the included time frame, the year with most events was 2018 (n=44), the year with least events was 2020 (n=12) if we exclude 2021 (given that only two-thirds of that year was included in our data set) (figure 2). Overall, BAT’s attendance at events slightly decreased over time, while PMI’s record shows an upward trend before a sharp decline in 2020 and 2021.

#### Location

Almost 80% of the events (170/213) were in high-income countries, while 5.2% (11/213) were in upper middle-income countries. Lower middle-income countries and low-income countries were not represented. The remaining 15% of events (32/213) took place online (8%, 17/213) or had an unclear location (7%, 15/213).

Over 40% of events (91/213) took place in the European region and around a third (74/213) in the Region of the Americas (71 of which were in the USA and Canada). Less than 8% of events (16/213) were held elsewhere: 9 (4.2%) in the Western Pacific, 3 (1.4%) in South-East Asia, 2 (0.9%) in the Eastern Mediterranean and 1 (0.5%) each in the African Region and Taiwan.

#### Fields

The events attended by BAT and PMI covered a diverse set of fields (see table 1). Toxicology was the most represented field (28.2%, 60/213), followed by medicine (11.7%, 25/213), biology (11.3%, 24/213) and chemistry (10.8%. 23/213). Within medicine almost half of the events focused on cardiovascular medicine. Other areas included pneumology, oncology, family medicine and neurology. Aerosol science (8.5%, 18/213) and tobacco/nicotine (5.2%, 11/213) were also represented in the data set. Other fields included dentistry, pharmaceutical science and computing.

**Table 1:** Events by field

Field*	n	%
Toxicology	60	28.2
Medicine	25	11.7
Cardiovascular	12	5.6
Pneumology	4	1.9
Oncology	2	0.9
Family medicine	2	0.9
Neurology	2	0.9
Ophthalmology	1	0.5
Gastroenterology	1	0.5
Medicine (not specified)	1	0.5
Biology	24	11.3
Chemistry	23	10.8
Aerosol science	18	8.5
Tobacco/nicotine	11	5.2
Dentistry	9	4.2
Pharmaceutical sciences	8	3.8
Computing	8	3.8
Experimental techniques	7	3.3
Risk analysis	5	2.3
Biomarkers	3	1.4
Food science	3	1.4
Harm reduction	3	1.4
Epidemiology	2	0.9
Occupational hygiene	2	0.9
Other	10	4.7
Not specified	5	2.3

*Some events covered multiple topics so the numbers and percentages do not add up to n=213 (100%).

#### Organiser

**Figure 3:**
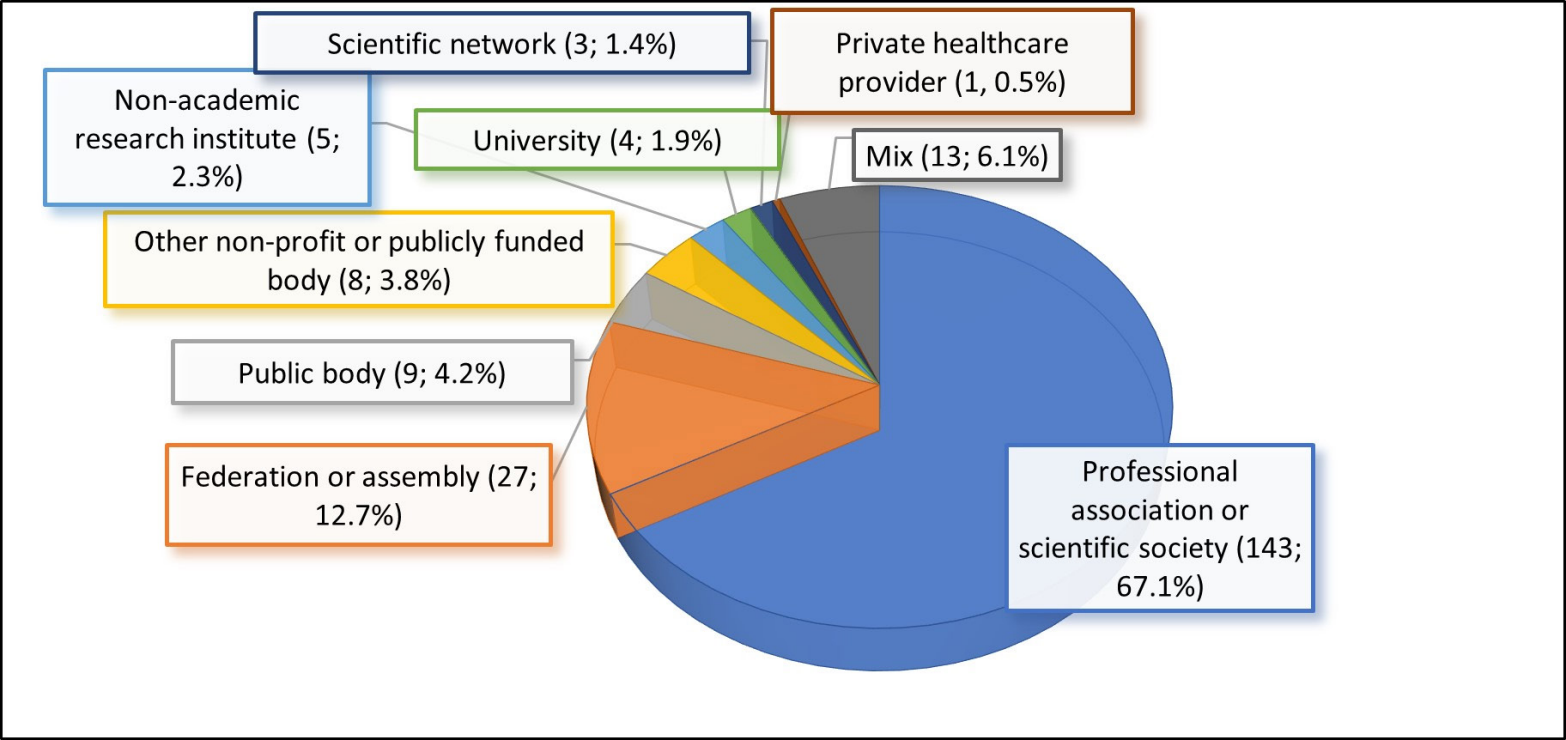
Event organisers by type.

Over two-thirds of the included events were organised by professional associations or scientific societies, including their working groups (figure 3). The next largest categories of organiser (12.7%) were federations or assemblies of professional associations or scientific societies. Over 4% were organised by a public body (six by the US Food and Drug Administration, two by the US Environmental Protection Agency and one by the British National Centre for the Replacement, Refinement and Reduction of Animals in Research).

Less common types of organisers were other non-profit or publicly funded organisations, universities, non-academic research institute, scientific networks and private health providers. A mixed set of organisers (eg, a public body and a university) ran 6.1% of included events.

#### Presentations and posters

According to the conference materials downloadable from BAT and PMI websites, BAT and/or PMI delivered talks at 65 (30.5%) and presented posters at 118 (55.4%) of the included events. BAT presented more materials (BAT: 50 presentations, 210 posters; PMI: 27 presentations, 146 posters) at more events (BAT: 43 events (presentations), 75 events (posters); PMI: 24 events (presentations), 60 events (posters); these numbers are higher than the total as both companies delivered presentations at two events and posters at 17 events).

### Findings from step 2: event websites and materials

#### Involvement in event sponsorship, exhibition and organising committee

We obtained data on tobacco industry involvement in 105 events from event websites or materials. Sponsors were documented for 88 events (83.8%), exhibitors were listed for 51 events (48.6%) and membership of organising committee was detailed for 48 events (45.7%) (see figure 1).

We found evidence of sponsorship from BAT and PMI or a subsidiary for nine events^53–61^ (10.2%, 9/88) (see online supplemental table 2). The level of sponsorship was only specified for one event: at the 41st Annual Meeting of the American College of Toxicology in 2020, PMI was a Gold Sponsor ($5000–$9999).^61^ BAT/BAT Science and PMI/PMI Science were exhibitors at three events^53 62 63^ (5.9%, 3/51). Finally, we found evidence of tobacco industry involvement in the organising committee for one event (2.1%, 1/48).^64^

## Discussion

This study reveals that BAT and PMI reported their involvement in a large number and diverse range of scientific events over the past decade. Following an increase of events after 2016, the number has fallen since 2019 with the strongest decline since 2020, likely linked to the COVID-19 pandemic which led to the cancellation of many events.^65 66^ BAT attended more events in the earlier years of the study period (2012–2015, most events in 2013 (n=22)), while PMI recorded more events in later years (2016–2021, most events in 2018 (n=34)). Events were mostly held in Europe and North America.

Several events focused on areas where tobacco industry interest and activity are well known, most notably toxicology, chemistry and aerosol research.^6^ The events on dentistry and biomarkers, for example, could reflect the increasing importance of newer nicotine and tobacco products.^67 68^ Similarly, attendance at pharmaceutical events could be explained by the tobacco industry’s efforts to link newer products to smoking cessation.^69^ For other areas such as food science the connection is less clear. It is particularly concerning that over 10% of the events were medicine related and that some medical and dental associations, federations and research institutes were allowing industry participation. In one instance, a PMI subsidiary directly sponsored an event organised by a medical society.^60^

Overall, involvement through sponsorship, exhibition or organising committee was rare. More common was the delivery of posters at over half of events and presentations at almost a third. This approach could be seen as an attempt to normalise tobacco industry presence in the academic setting, enabling the industry to present itself as a legitimate stakeholder in evidence production and evidence-based decision-making.^1^

While most events were organised by professional associations or federations, some were organised by publicly funded bodies which could indicate a violation of Article 5.3 of the WHO Framework Convention on Tobacco Control (FCTC) that prohibits ‘unnecessary interactions’ with the industry.^70^ Eight of the nine events were organised by a US public body; the US government is yet to ratify the WHO FCTC.

Our study has a number of limitations. First, we relied on information provided by BAT and PMI and could not verify the completeness of data. When we collected the data (last updated in September 2021), the companies only recorded events attended between May 2012 (BAT)/July 2013 (PMI) and October 2020 (BAT)/September 2021 (PMI). We also found an example of incomplete reporting: in one event listed only in the BAT database, we found that the event’s website also listed PMI Japan as a sponsor.^59^ Second, we did not capture where individuals or organisations funded by the tobacco industry were involved in events. For example, we repeatedly came across sbv (Systems Biology Verification) IMPROVER,^54 62 71 72^ a project led and funded by PMI.^73^ PMI did not list these involvements on its website. Given the tobacco industry’s track record of using front groups and concealing these activities,^74^ as well as the variety of known third parties working on behalf of the industry,^75^ it is possible that our study significantly underestimates the involvement of the tobacco industry in scientific events.

Third, we could not obtain relevant information regarding sponsorship, exhibitors and organising committees for many events. This means that our findings could particularly underestimate this element of tobacco industry involvement. Furthermore, the information about the organising committee was often very limited: while members were usually listed, affiliations were often missing which did not allow us to detect potential industry involvement. A final limitation is that our analysis of event websites focused on tobacco industry presence as sponsor, exhibitor or within the organising committee and did not examine whether event organisers had policies to manage relationships with sponsors or conflicts of interests within the organising committee.

Despite these limitations which likely led to an underestimation of industry participation in scientific events, our findings underpin the need for the public health community to address industry presence at and involvement in scientific events. The increase in tobacco industry attendance at scientific events should be seen as part of what Briggs and Vallone have labelled tobacco industry’s "renewed assault on science".^76^ It is well established that corporations seek both to normalise their presence in academia and to use science as a tool for renormalisation.^1^ Their influence on science is ultimately about maximising profit in part by establishing industry products as solutions.^1^ As such, failure to address this issues could—even if unwittingly—contribute to tobacco companies’ agenda.

Hitherto, actions taken by conference organisers, journal editors and funders appear limited to those with greater experience and understanding of the tobacco industry. Our work highlights the many other fields which could benefit from the robust protections required to ensure science in the public interest.

Past efforts to limit industry participation also need to be critically evaluated: for example, while the World Conference on Tobacco or Health excludes those working for or supported by the tobacco industry since 1990,^19 20^ the 2021 SRNT decision only banned employees of the tobacco companies and "ENDS [e-cigarette] companies that are wholly or partially owned by the traditional tobacco product manufacturers" from membership and from attending the Society’s annual conference.^22 77^ Those consulting for tobacco or ENDS industries remain eligible for SRNT membership^22^ which is concerning given the industry’s track record of working through consultants.^8 40 78^

When organisers decide whether to interact with commercial actors, consideration should go well beyond individual conflict of interest—the assessment of the public health harms of the actor’s products and institutional/structural conflicts of interest is essential.^79 80^ We also encourage all organisers to publicly report event sponsors, exhibitors and members of organising committees, including their affiliation and conflicts of interest, in an accessible way to enhance transparency.

Further research could broaden this work by also assessing the involvement in events of other actors, including companies supplying the tobacco industry as well as tobacco industry allies. Furthermore, the relationships between professional associations and industry could be further explored, and the policies and funding of organisations whose events have been attended by the tobacco industry could be examined. Such work has so far mostly focused on the pharmaceutical industry.^81^ Additional research could also assess the content of tobacco industry posters and presentations. Finally, building on existing work into tobacco companies’ strategic use of social media to shape their public identity and influence policymaking,^82^ one could examine how tobacco companies use their science Twitter accounts (eg, BAT Science, PMIScience) to gain more insights into their efforts to communicate with the scientific community.

## Conclusion

This is the first study to systematically analyse the tobacco industry’s involvement in scientific events. Drawing on information available on tobacco companies’ websites and supplementary searches, we found that BAT and PMI representatives attended a wide range of events. More action against the tobacco industry’s use of scientific fora is needed. This could include encouraging conference organisers to adopt policies regarding industry involvement and funding as well as raising awareness among scientists beyond the public health community.

## Data Availability

Data are available upon reasonable request.
