# Supplementary material for: Seeking to be seen as legitimate members of the scientific community? An analysis of British American Tobacco and Philip Morris International’s involvement in scientific events
Source: Tob Control. 2023 Feb 3;33(4):464–71. doi: 10.1136/tc-2022-057809 (PMC11228186; doi:10.1136/tc-2022-057809)
Supplement: Supplementary data [file tc-2022-057809supp001.pdf]

Supplemental material

Table 1: Analysis of conference websites and materials: Manual for the coders

**Question**	**Answer (where different options, delete irrelevant ones)**	**Additional information**	**Instructions**
Before you start, please ***double check that you are looking at the right website:*** *some conference websites contain information on the annual conferences from several years, so please double check that you are in the correct section of the website.*			
**General information**			
1. Name of the conference			Copy and paste
2. Year of the conference			Copy and paste
3. Location			
4. Weblink used for data extraction			Copy and paste
4.How was the website identified?	1=Link provided by PMI<br>2= Google search<br>3= PMI link available but page identified through Google search		PMI provides links to websites of conference. This could be the starting point. Otherwise, if the link is not available or does not work, put the name of the conference in Google.
5.Is the website still active?	Yes/No		If No, you can stop and not proceed with the following questions.
**Conference sponsors**			
6.Was PMI listed among the conference sponsors?	1=Yes<br>2=No<br>3=Information on sponsors not available on the website	If yes, link:	There is usually a dedicated section of the website called “Supporters” or “Sponsors”. But it needs to be explicit that it’s about financial support/ sponsorship.

Matthes BK, *et al. Tob Control* 2024; 33:464–471. doi: 10.1136/tc-2022-057809

6a. If yes, collect information on the sponsorship tier where available (e.g., Bronze, Silver, Gold, Platinum)	Free text	A pdf or other document is saved if available, otherwise, a screenshot is taken. The evidence is saved in the “evidence” folder.	Check whether PMI or BAT are in this section and if available, collect also the tier PMI/BAT are in and the amount (e.g. Platinum ($5,000–$9,999))
6b. If yes, copy and paste any other information.	Free text		
7. Was BAT listed among the conference sponsors?	1=Yes<br>2=No<br>3=Information on sponsors not available on the website	If yes, link:<br><br>A pdf or other document is saved if available, otherwise, a screenshot is taken. The evidence is saved in the “evidence” folder.	
7a. If yes, collect information on the sponsorship tier where available (e.g., Bronze, Silver, Gold, Platinum)	Free text		
7b. If yes, copy and paste any other information	Free text		
**Organising committee**			
8.Were PMI employees involved in the organising committee?	1=Yes<br>2=No<br>3=Information on organising committee not available on the website<br><br>If yes, please collect the name of the employee and its role within PMI.	If yes, link:<br><br>A pdf or other document is saved if available, otherwise, a screenshot is taken. The evidence is saved in the “evidence” folder.	The website usually contains a section called “organising committee” or “program committee”. Names and affiliations of the members are provided and you should check them to see if anyone is affiliated with PMI or BAT.

Matthes BK, *et al. Tob Control* 2024; 33:464–471. doi: 10.1136/tc-2022-057809

9.Were BAT employees involved in the organising committee?	1=Yes<br>2=No<br>3=Information on organising committee not available on the website<br><br>If yes, please collect the name of the employee and its role withim BAT.	If yes, link:<br><br><br>A pdf or other document is saved if available, otherwise, a screenshot is taken. The evidence is saved in the “evidence” folder.	
**Conference exhibitors**			
10.Was PMI among the conference exhibitors?	1=Yes<br>2=No<br>3=Information on conference exhibitors not available on the website	If yes, link:<br>A pdf or other document is saved if available, otherwise, a screenshot is taken. The evidence is saved in the “evidence” folder.	Check the section called “Exhibitors” or “Booth” or “EXPO” and look for PMI or BAT presence. This is not about poster or oral presentations.
11.Was BAT among the conference exhibitors?	1=Yes<br>2=No<br>3=Information on conference exhibitors not available on the website	If yes, link:<br>A pdf or other document is saved if available, otherwise, a screenshot is taken. The evidence is saved in the “evidence” folder.	
**Any comments**			
Please write here any additional relevant information you found on the conference website or any doubt you had while filling in the form.			

Matthes BK, *et al. Tob Control* 2024; 33:464–471. doi: 10.1136/tc-2022-057809

Table 2: BAT and PMI's involvement as sponsors, exhibitors and in organising committees

Year	Event (organiser)	Sponsor		Exhibitor		Organising committee	
		BAT	PMI	BAT	PMI	BAT	PMI
**2013**	Matrix Challenging Analytical Science (Chromatographic Society)[1]	BAT		BAT			
**2013**	15th Annual Congress of the European Society for Alternatives to Animal Testing (EUSAAT)[2]	BAT					
**2015**	Young Scientists and Researchers Environmental & Food Analysis SIG Meeting (EFASIG) [3]					BAT R&D (with British Mass Spectrometry Society (BMSS))	
**2017**	Eurotox 2017 (European Societies of Toxicology)[4]			BAT Science*			
**2018**	3rd Conference of the International Society for Plant Molecular Farming (ISPMF)[5]	BAT Science*					
**2018**	7th Annual Meeting of the American Society for Cellular and Computational Toxicology (ASCCT)[6]	BAT					
**2018**	SwissPLANT (Swiss Plant Science Web) [7]		PMI				
**2019**	26 Congresso Nazionale (Collegio dei Docenti Universitari di discipline Odontostomatologiche)[8]	BAT Science*					
**2019**	6th Congress of Asian College of Neuropsychopharmacology (AsCNP)[9]	BAT Japan**					
**2019**	29th Congress of the Mediterranean League of Angiology and Vascular Surgery (MLAVS)[10]		Philip Morris Zagreb***				
**2019**	XXIII Congresso Nazionale COI-AIOG (Cenacolo Odontostomatologico Italiano – Associazione Italiana di Odontoiatria Generale)[11]				PMI Science****		
**2020**	41st Annual Meeting of the American College of Toxicology (ACT 2020)[12]		PMI Science****				
**Total**		6	3	2	1	1	0

**BAT Science is BAT's research and development department*[13], ***BAT Japan is a subsidiary of BAT.*[14], ***Philip Morris Zagreb is a subsidiary of PMI.[15], ****PMI Science is operated by PMI.[16]

Matthes BK, *et al. Tob Control* 2024; 33:464–471. doi: 10.1136/tc-2022-057809

References

1. Chromatographic Society. Matrix Challenging Analytical Science - Symposium. 2013. https://web.archive.org/web/20220411151225/https://www.labbulletin.com/downloads/20130422/download, accessed on 11/04/2022.
2. European Society for Alternatives for Animal Testing. EUSAAT 2013 - Sponsors & supporters. 2013. https://web.archive.org/web/20220411151822/https:/www.eusaat-congress.eu/index.php/congress/2013/sponsors, accessed on 11/04/2022.
3. British Mass Spectrometry Society/ BAT. EFASIG2015: Young Scientists and Researchers Environmental & Food Analysis SIG Meeting. 2015. https://web.archive.org/web/20220411154622/https:/www.rsc.org/events/detail/19297/young-scientists-and-researchers-environmental-and-food-analysis-sig-meeting, accessed on 11/04/2022.
4. European Societies of Toxicology. Eurotox 2017 - Exhibiton. 2017. https://web.archive.org/web/20220411154043/https:/www.eurotox2017.com/exhibition/, accessed on 11/04/2022.
5. International Society for Plant Molecular Farming. The 3rd Conference of the International Society for Plant Molecular Farming - Book of Abstracts. 2018. http://web.archive.org/web/20220411152208/https:/core.ac.uk/download/pdf/292466514.pdf, accessed on 11/04/2022.
6. American Society for Cellular and Computational Toxicology. 7th Annual Meeting of the ASCCT. 2018. https://web.archive.org/web/20210613013407/https:/www.ascctox.org/meeting/38, accessed on 11/04/2022.
7. Swiss Plant Science Web. SwissPLANT 2018. 2018. https://web.archive.org/web/20220411153341/https:/swissplantscienceweb.unibas.ch/fileadmin/user_upload/swissplantscienceweb/Event/Swissplant/swissplant2018_booklet_web.pdf, accessed on 11/04/2022.
8. Collegio de Docenti Universitari Di Discipline Odontostomatologiche. 26° Congresso Nazionale. 2019. https://web.archive.org/web/20220411152814/http:/www.collegiouniversitariodiodontostomatologia.it/images/congressinazionali/programma2019.pdf, accessed on 11/04/2022.
9. Asian College of Neuropsychopharmacology. 6th Congress of ASCNP - Program&Abstract Book. 2019. https://web.archive.org/web/20220411153140/https:/www.ascnp.org/event/6th_ascnp_congress_2019-lba-award-compressed.pdf, accessed on 11/04/2022.
10. Mediterranean Legue of the Angiology and Vascular Surgery. The XXIX Congress of MLAVS - Sponsors. 2019. https://web.archive.org/web/20220411161131/https:/corporate.btravel.pro/en/congresses/mlavs/, accessed on 11/04/2022.
11. Cenacolo Odontostomatologico Italiano. XXIII Congresso Nazionale. 2019. http://web.archive.org/web/20220415204854/https://www.aiditalia.it/wp-content/uploads/2019/03/Brochure-XXIII-Congresso-Nazionale-COI-AIOG.pdf, accessed on 11/04/2022.
12. American College of Toxicology. Annual Meeting Supporters. 2020. https://web.archive.org/web/20220411153559/https://www.actox.org/am/am2020/sponsorlist.asp, accessed on 11/04/2022.
13. BAT Science. [Twitter]. n.d. https://web.archive.org/web/20220601104006/https://twitter.com/BAT_Sci?ref_src=twsrc%5Egoogle%7Ctwcamp%5Eserp%7Ctwgr%5Eauthor, accessed on 01/06/2022.
14. Bloomfield M, Hiscock R, Mehegan J, et al. The Tobacco supply chains Database. 2021. https://tobaccotactics.org/supply-chain/, accessed on 02/03/2022.
15. PMI. Croatia. n.d. http://web.archive.org/web/20220601104428/https://www.pmi.com/markets/croatia/en/overview, accessed on 01/06/2022.
16. PMIScience.com. About PMI. n.d. http://web.archive.org/web/20220601105449/https://www.pmiscience.com/en/, accessed on 01/06/2022.

Matthes BK, *et al. Tob Control* 2024; 33:464–471. doi: 10.1136/tc-2022-057809
